# The effect of temporary hemiepiphysiodesis in the treatment of skeleton immature posttraumatic genu angular deformity: a retrospective study of 27 cases

**DOI:** 10.1186/s13018-019-1426-0

**Published:** 2019-11-21

**Authors:** Jing Ding, Ting Zhu, Fang-chun Jin, Zhen-kai Wu, Hai Li

**Affiliations:** 1 Department of Pediatric Orthopaedics, Xinhua Hospital, School of Medicine, Shanghai Jiao Tong University, No. 1665, Kongjiang Road, Shanghai, 200092 China; 2 Clinical Research Unit, Xinhua Hospital, School of Medicine, Shanghai Jiao Tong University, Shanghai, 200092 China

**Keywords:** Temporary hemiepiphysiodesis, Genu fracture, Angular deformity

## Abstract

**Background:**

The purpose of this study was to evaluate the effect, rate of angular correction, and complications of temporary hemiepiphysiodesis (TH) in the treatment of skeleton immature posttraumatic genu angular deformity.

**Methods:**

We retrospectively reviewed the records of 27 patients undergoing temporary hemiepiphysiodesis for the management of posttraumatic genu angular deformity. Based on the data from these patients, the rate of correction, effect of correction, length of the lower limbs, and complications were used as the outcome measures.

**Results:**

Outcome measurements were obtained from a chart review of medical records that included information about clinical evaluations. Fifteen boys and 12 girls, with an average age of 6.3 years, were included in the study. The average follow-up was 3.8 years (range, 1.9 to 5.9 years) after surgery. Complete correction was obtained in 24 patients, while partial correction was obtained in 3 patients. The mean rate of angular correction was 8.41°/year in distal femur and 15.19°/year in proximal tibia. One patient had recurrence of genu valgum. No leg length discrepancy was found in our patients.

**Conclusion:**

Temporary hemiepiphysiodesis is a simple, effective, reliable, and reproducible method for the treatment of posttraumatic genu angular deformity, with fewer complications than osteotomy. Nevertheless, it is important to follow the rebound patient closely until skeletal maturity in our future work.

## Background

Fractures around the knee (distal femur or proximal tibia fractures) are common types of child fractures. Posttraumatic genu angular deformity has been an unpredictable outcome for patients and is difficult to cure [1]. Four decades ago, this deformity was treated with a brace, but follow-up results have shown no substantial effect on slowing the progression of the deformity [2]. Zionts et al. thought that the limbs had spontaneous correction and compensatory ability, and the slight angular deformity (< 15°) was observed without any treatment [3]. The genu varus or valgum in some cases may lead to mechanical axis deviation, genu articular surface unevenness, and increased intra-articular pressure and may finally result in secondary genu articular degeneration [4, 5]. Corrective osteotomy is an effective treatment, but lead to severe complications, such as compartment syndrome, vascular injury, nerve injury, deep tissue infection, and bone nonunion. In addition, corrective osteotomy requires a long postoperative recovery time. Postoperative recurrence requires reoperation, which is painful for patients [6]. Temporary hemiepiphysiodesis (TH) is an attractive option in correcting the angular deformity, with advantages over other techniques, including easier operation, lesser trauma, and fewer complications. Compared with traditional hemiepiphysiodesis, TH has a better prospect of application, because it does not injure the epiphyseal plate and can be repeated [7, 8]. Although some articles have reported on the use of this technique for correcting angular deformity, there are fewer reports about the effect of treatment in skeleton immature posttraumatic genu angular deformity [9, 10]. The purpose of this study was to evaluate the effect, rate of angular correction, and complications of TH in the treatment of this kind of patients.

## Methods

**Fig. 1:**
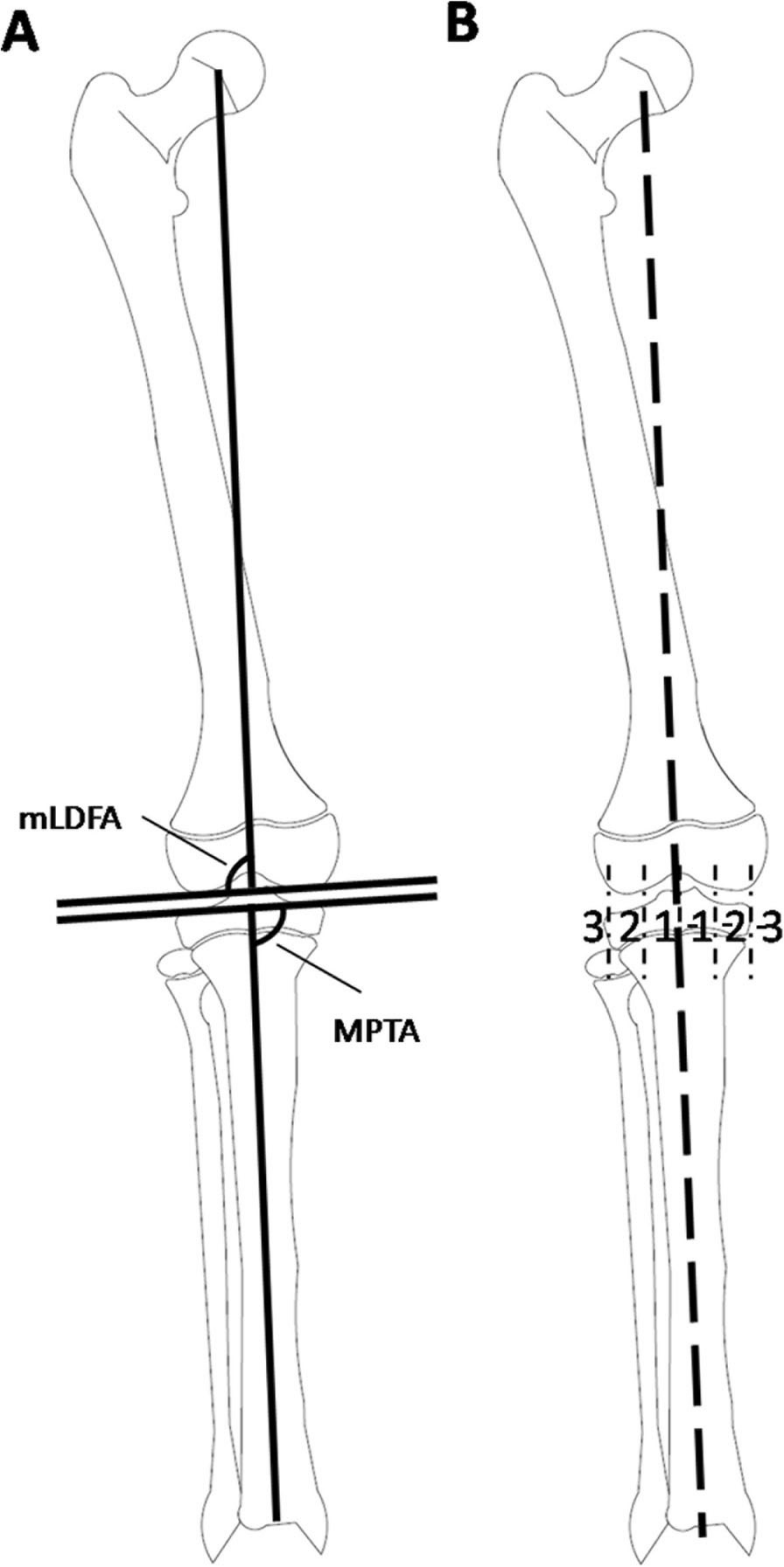
Angular measurement and mechanical axis zones. (**a** mLDFA: the lateral mechanical angle of the distal femur. MPTA: the medial angle of the proximal tibia. **b** Dividing the knee into 6 areas, the mechanical axis (dotted line) in zone 1 (lateral zone) or – 1 (media zone) was considered fully corrected, in zone 2 (lateral zone) or – 2 (media zone) was considered partially corrected, and in zone 3 (lateral zone) or – 3 (media zone) was considered uncorrected)

A single-center, retrospective, cohort study was performed in our study. Cases with initial temporary hemiepiphysiodesis from January 2010 to July 2015 were observed and reviewed, with a minimum 18 months of clinical and radiographic follow-up. Inclusion criteria were (1) angular deformity as a result of isolated distal femur and proximal tibia fractures without any concomitant disease, (2) growth plate still open after implant removal, (3) follow-up time > 18 months, and (4) mechanical axis in zone − 3 or 3 (Fig. 1). Exclusion criteria were (1) epiphyseal plate involved in trauma resulting in a bridging callus formation, (2) accompanying skeletal dysplasia or metabolic disease, (3) benign neoplasm of bones, (4) other surgical history in the knee and its surrounding area related to the growth plate, and (5) unavailable whole full-length anteroposterior standing radiographs pre-operation and post-operation. A total of 27 patients accorded with the inclusion criteria. There were 15 males and 12 females in this series, with an average age of 6.3 years (range, 2.5 to 9.7 years). The average follow-up was 3.8 years (range, 1.9 to 5.9 years) after surgery. All procedures were accomplished according to the methods of Stevens [8] by pediatric orthopedist who had been trained in TH. In our management, immobilization was not required, early-bearing walk was encouraged in 2 days after surgery, and the patients can return to school without any sports limitation in 2 weeks after surgery. Routine follow-up was arranged at 3 month intervals. Once full correction of the deformity and neutralization of the mechanical axis were achieved, the plate and screws were removed.

## Outcome measurement

The outcome measurement was in accordance with the methods of Stevens [11], dividing the knee into 6 areas (zones 1, 2, 3, − 1, − 2, and − 3), and before removing the inner fixation, assessing the change of the mechanical axis. The mechanical axis went across zone 1, − 1 was considered fully corrected; zone 2, − 2 partially corrected; and in zone 3, − 3 uncorrected (Fig. 1). When the mechanical axis went across zone 1 or − 1, the plate and screw removal are scheduled accordingly. Otherwise, the angular deformity would be overcorrected with the continuing growth of femur and tibia. After implant removal, the rebound was defined as the mechanical axis going back to zone 3 or − 3 again. The rate of correction was calculated as the difference in mechanical lateral distal femur angle (mLDFA) and the medial proximal tibia angle (MPTA) between pre-operation and removing inner fixation, divided by the reserving time of inner fixation in vivo. A difference in length of over 2 cm indicated dual lower limbs length inequality and under 2 cm indicated no difference in the length of the dual lower limbs. Follow-up after the operation consisted of assessing for deformity recurrence and other complications. All imaging data were obtained and measured by PACS (Picture Archiving and Communication Systems, UniWed, 6.1, EBM Technologies, Shanghai).

## Results

**Fig. 2:**
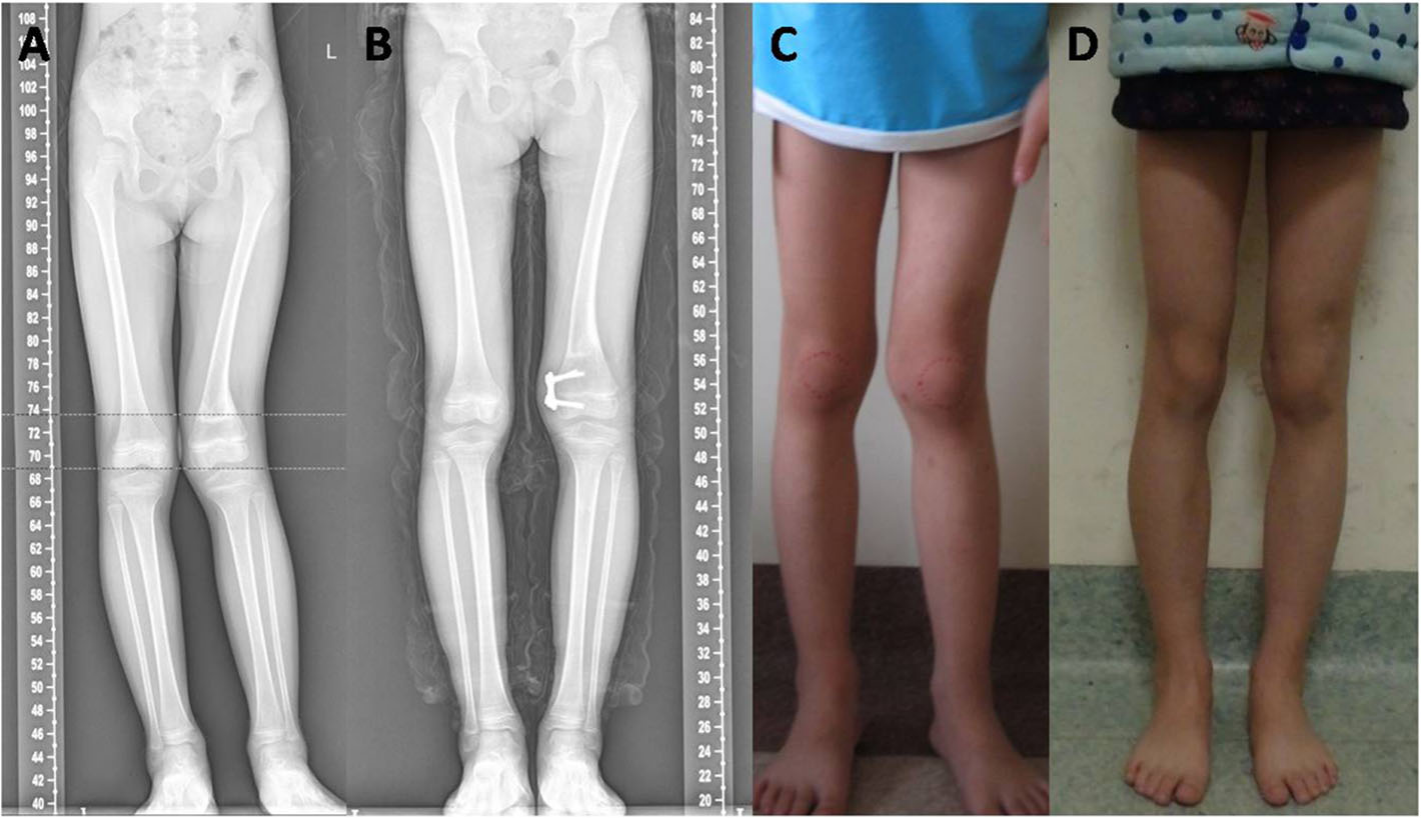
Comparison of full-length anteroposterior standing radiographs pre-operation and before removing the inner fixation in a girl about 6 years of age, with external photos. (**a** pre-operation X radiograph, **b** removing inner fixation X radiograph, **c** pre-operation external photo, **d** external photos before removing inner fixation)

**Fig. 3:**
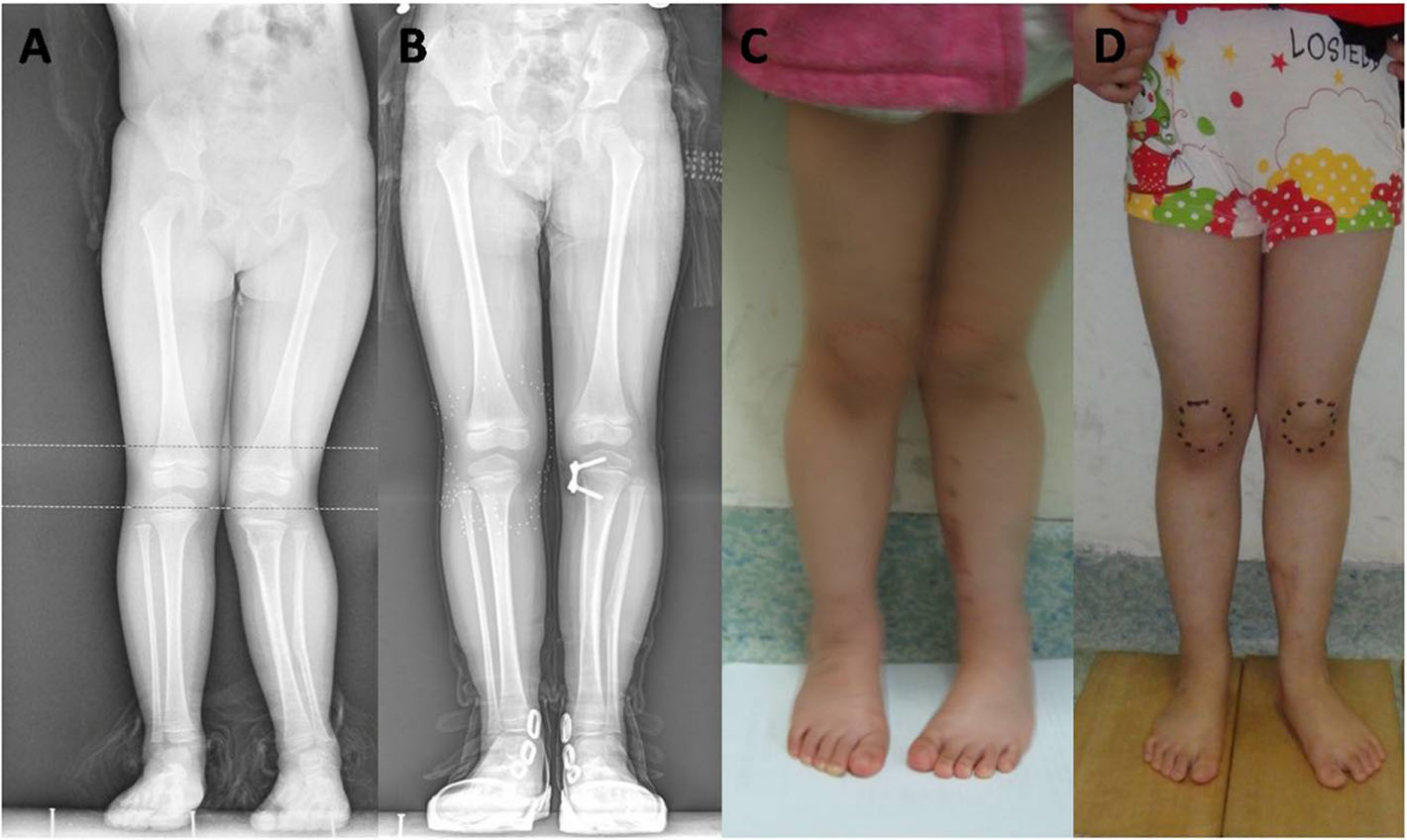
Comparison of full-length anteroposterior standing radiographs pre-operation and before removing the inner fixation in a 3-year-old girl, with external photos. (**a** pre-operation X radiograph, **b** removing inner fixation X radiograph, **c** pre-operation external photo, **d** external photos before removing inner fixation)

The outcome measurement was obtained from the chart review of medical records and clinical evaluation at the clinics (Table 1). Full correction was obtained in 24 patients (Figs. 2 and 3), while it was not achieved in 3 patients. The average operative time was 35 min (range 27 to 58 min), and the average bleeding lost was 11.5 ± 2.9 ml, 13.7 ml for the distal femur, and 10.4 ml for the distal femur. The proximal tibia angle correction was 15.19°/year, and the distal femur angle correction was 8.41°/year. The average time of internal fixation reserve was 1 year (0.9–1.9 years). Infection and delayed union of fracture were not found. There was one rebound deformity in 2 years post-operation, and the patient had reoperation. Other patients did not have a reoccurrence at the time of follow-up. All patients were found to have equal limb length. At the time of follow-up, no patients had limited joint mobility or epiphyseal plate closure.

**Table 1:** The clinic characteristic of patients

	Clinic characteristic
Number of case	27 (15 m/12 ft)
Average age	6.3 years (2.5–9.7)
Surgical site	9 in distal femur and 18 in proximal tibia
Surgical time	35 min (27–58)
Follow-up time	3.8 years (1.9–5.9)
Average time of implant reserve	1.0 years (0.9–1.9)
Rate of correction in distal femur	8.41°/year
Rate of correction in proximal tibia	15.19°/year
Rate of full correction	0.89 (24/27)

## Discussion

Fractures around the knee and genu angular deformity of children are familiar in the clinic, although the mechanisms of their association are not definite. Some mechanisms might relate to asymmetric growth of the epiphyseal plate stimulated by trauma, tractusiliotibialis hitched epiphyseal plate, or force distribution on the ligaments around the knee not being uniform after cast immobilization [12, 13]. For decades, the deformity has been difficult to cure only with above knee brace [2]. Zionts et al. thought that because of children’s spontaneous correction and compensatory ability, the nihilistic approach could be adopted with patients [3]. However, Skak thought that self-adjustment was not reliable according to follow-up results, and there was residual deformity, which might induce genu degeneration [14]. Although the deformity could be fully corrected through different kinds of osteotomy, these methods resulted in many complications, including severe injury to children, and were expensive [6]. Moreover, osteotomy was not an appropriate first choice for treatment because children’s epiphyseal plates were unclosed and the deformity might recur.

In 1993, Phemister et al. [15] introduced epiphysiodesis as a therapy for genu angular deformity of children. The surgery corrected the deformity by arresting the growth on one side of the epiphyseal plate, while permitting normal growth on the other side. Bowen et al. [16] presented the technique of percutaneous epiphysiodesis under fluoroscopic guidance to correct the deformity. The Bowen’s technique is a lesser invasive surgery. Both of these surgeries, however, were permanent, so the timing of the operation was very important. At present, there is not a reliable method to predict the growth potential of epiphyseal plate, so undercorrection or overcorrection is a common problem using these techniques. Stainless steel U-shaped epiphyseal nails designed by Blount [17] limited the growth of specific area temporarily and reserved the growth potential. When the inner fixations were removed, the epiphyseal plate was still growing normally. This surgery was named temporary hemiepiphysiodesis. Compared with permanent epiphysiodesis, it did not require a strict operation time and could be used repeatedly. In 2007, Stevens et al. reported that an improved 8-plate replacing traditional U-shaped epiphyseal nails had an ideal effect in angular deformity patients who accepted TH.

Although there are many reports about the use of TH to treat lower limb angular deformity caused by all kinds of reasons [10, 18], few of these reports specifically describe the treatment of fracture accompanied by genu deformity. Our study addressed whether or not this method is simple, reliable, repeatable, and has complications for treating these kinds of patients. We followed 27 knee joint fracture patients: 9 with distal femur fractures and 18 with tibial proximal fractures. All patients were treated with inner fixation or plaster fixation, and all had gradual appearance of genu angular deformity. Before the operation, if the difference between mLDFA and standard mLDFA (88°) of the patients was over 15° and the mechanical axis was in zone 3 or − 3, we chose the epiphyseal plate of the distal femur as the operative position. When the difference between MPTA and standard MPTA (87°) of the patients was over 15° and the mechanical axis was in zone 3 or − 3, we chose the proximal tibia as the operative position. All operations were accomplished by two pediatric orthopedists who had professional training in TH. The operative process was simple, took little time to perform, and resulted in less bleeding than other methods. The operation was safe and resulted in no complications of infection, inner-fixation failure, or knee joint limitation. Patients’ ability to walk soon after the procedure alleviated their pain and was advantageous to recovery of function. The outcome demonstrated that complete correction was obtained in 24 patients and the inner fixation was removed, while partial correction was achieved in 3 patients. These results were in accordance with Stevens [10], which indicated that treatment of this deformity with TH was valid. The average corrective time was 1 year, and we found that the rate of correction in femur or tibia was very close to it in idiopathic genu varus or valgum [11]. This suggested that the growth potential was obviously not affected by the trauma. Despite their differences, the fractures complicated by genu angular deformity and genu valgus had no difference in effect of the treatment with TH. Contrary to some studies, our results showed that the average rate of correction for the tibia was faster than that for the femur [7–9]. These results might be correlative with the different average age of the two groups of patients. The average age of the tibia group was 3.8 years, whereas that of the femur group was 8.6 years. Therefore, we speculated the rate of correction was faster in younger patients than older ones. These results were consistent with Paley [18]. However, caution should be taken when using TH for older patients whose epiphyseal plate is nearly closed. One patient’s deformity recurred after the internal fixation was removed. The patient suffered TH again, and the angular deformity has an 8° (mLDFA) improvement at follow-up. At that time, the internal fixation was not removed. Considering that the epiphyseal plate of some patients may not be closed at follow-up, possibly indicating recurrence, we suggest that these patients continue to be followed up until the epiphyseal plate closes. If the deformity does recur, then the same operation can be performed again. We did not find that the two lower limbs had different lengths as a result of the fracture or after TH.

There were several limitations to our study. First, this was a retrospective series over the course of 6 years with low evidence level. Second, the sample size was small due to the rare rate of posttraumatic genu angular deformity. Third, the different age as a confounder factor may contribute to different rate of correction. It might be the reason why some cases was not corrected completely. Fourth, the epiphyseal plate was still growing in some cases, so the rate of recurrence was supposed to be higher.

## Conclusion

Temporary hemiepiphysiodesis is a simple, effective, reliable, and reproducible method for the treatment of posttraumatic genu angular deformity, with fewer complication rates than osteotomy. We found no obvious length inequality of the dual lower limbs. Nevertheless, it is important to follow the rebound patients closely until skeletal maturity in our future work.

## Data Availability

The datasets used and analyzed during the current study are available from the corresponding author on reasonable request.

## Abbreviations

LDFA: Lateral distal femur angle
MPTA: Media proximal tibia angle
PACS: Picture Archiving and Communication Systems
TH: Temporary hemiepiphysiodesis

## References

[1] Jordan SE, Alonso JE, Cook FF (1987). The etiology of valgus angulation after metaphyseal fractures in children. J Pediatr OrthopR.

[2] Jackson DW, Cozen L (1971). Genu valgum as a complication of proximal tibial metaphyseal fractures in children. J Bone Joint Surg..

[3] Zionts LE, MacEwen GD (1986). Spontaneous improvement of post-traumatic tibial valga. J Bone Joint SurgR.

[4] Tarr RR, Resnick CT, Wagner KS (1985). Changes in tibiotalar joint contact following experimentally induced tibial angular deformity. Clin Orthop..

[5] Stevens P, MacWilliams B, Mohr A (2004). Gait analysis of stapling for genu valgum. J Pediatr OrthopR.

[6] Steel H, Sandrow R, Sullivan P (1971). Complications of tibial osteotomy in children for genu valgum or varum. J Bone Joint Surg..

[7] Bowen JR, Leahey JL, Zhang ZH (1985). Partial epiphysiodesis at the knee to correct angular deformity. Clin Orthop..

[8] Stevens PM (2007). Guided growth for angular correction: a preliminary series using a tension band plate. J Pediatr Orthop.

[9] Raab P, Wild A, Seller K, Krauspe R (2001). Correction of length discrepancies and angular deformities of the leg by Blount’s epiphyseal stapling. Eur J Pediatr.

[10] Stevens PM, Pease F (2006). Hemiepiphysiodesis for posttraumatic tibial valgus. J Pediatr Orthop.

[11] Stevens PM, Maguire M, Dales MD (1999). Physeal stapling for idiopathic genu valgum. J Pediatr Orthop.

[12] Herring JA (1981). Post-traumatic valgus deformity of the tibia. J Pediatr Orthop..

[13] Houghton GR, Rooker GD (1979). The role of periosteum in the growth of long bones. J Bone Joint Surg..

[14] Skak SV (1982). Valgus deformity following proximal tibial metaphyseal fractures in children. Acta Orthop Scand..

[15] Phemister DB (1933). Operative assessment of longitudinal growth of bones in the treatment of deformities. J Bone Joint Surg.

[16] Bowen JR, Johnson WJ (1984). Percutaneous epiphysiodesis. Clin Orthop.

[17] Blount WP, Clarke GR (1949). Control of bone growth by epiphyseal stapling: a preliminary report. J Bone Joint Surg[Am].

[18] Paley D (2005). Principles of deformity correction. Chapter 20.

